# Combating Disparities in a Pandemic: Increasing Dissemination of Coronavirus Disease 2019 Resources in Spanish

**DOI:** 10.1097/pq9.0000000000000744

**Published:** 2024-07-10

**Authors:** Romina L. Barral, Nicholas A. Clark, Fernando Zapata, Lines M. Vargas Collado, July Jean Cuevas, Cristina Fernandez

**Affiliations:** From the * Division of Adolescent Medicine Children’s Mercy Hospital and Clinics, University of Missouri-Kansas City School of Medicine, Kansas City, Mo.; University of Kansas Medical Center, School of Medicine, Kansas City, Kans.; † Division of Pediatric Hospital Medicine, Department of Pediatrics, Children’s Mercy Hospital and Clinics, University of Missouri-Kansas City School of Medicine, Kansas City, Mo.; ‡ Division of Gastroenterology, Department of Pediatrics, Children’s Mercy Hospital and Clinics, University of Missouri-Kansas City School of Medicine, Kansas City, Mo.; § Division of Neurology, Department of Pediatrics, Children’s Mercy Hospital and Clinics, University of Missouri-Kansas City School of Medicine, Kansas City, Mo.; ¶ Division of Developmental and Behavioral Sciences, Department of Pediatrics, Children’s Mercy Hospital and Clinics, University of Missouri-Kansas City School of Medicine, Kansas City, Mo.; ∥ Division of Weight Management, Department of Pediatrics, Children’s Mercy Hospital and Clinics, University of Missouri-Kansas City School of Medicine, Kansas City, Mo.

## Abstract

**Introduction:**

Disparities exist in access to coronavirus disease 2019 (COVID-19)-related health information. We aimed to close a gap in online traffic between English and Spanish COVID-19-related health information on our institution’s publicly-facing website by 50% within ten months.

**Methods:**

We used A3 improvement methodology. Outcome measures were the mean monthly difference between English and Spanish COVID-19 online traffic vis-a-vis (1) total webpage views and (2) unique webpage visits. Process measures were stratification of outcome measures by language. Plan-Do-Study-Act cycles included: Recurring advertisements on a local Spanish television station disseminating up-to-date COVID-19 information, including our institution’s Spanish COVID-19 online resources, incorporation of QR codes into clinic discharge paperwork linking to institutional Spanish COVID-19 resources, and leveraging social media to expand reach. Control charts assessed impact over time.

**Results:**

There were 1,226,196 total webpage views (369,983 Spanish; 856,213 English) and 1,065,536 unique webpage visits (350,518 Spanish; 715,018 English). Both outcome measures displayed sustained, special cause improvement from a mean monthly difference of 25,397 to 11,321 webpage views (55.4% reduction, June 2021) and 25,066 to 7080 unique webpage visits (71.8% reduction, February 2021) corresponding to special cause improvements in process measures. Improvements were not temporally associated with an intervention but coincided with emergency use approval of the COVID-19 vaccine for children aged 12–15 years (May 2021).

**Conclusions:**

Although our interventions did not directly show improvements in our measures, we noted increased page views of Spanish COVID-19-related health information on our institution’s publicly-facing website in times of high demand for linguistically appropriate services, including pediatric vaccine roll-out.

## INTRODUCTION

Worldwide, the coronavirus disease 2019 (COVID-19) pandemic magnified already existing health disparities. Eliminating health disparities among those who experience a disproportionate burden of disease will significantly improve the future of our nation’s health.^1^ Eliminating health disparities, achieving health equity, and attaining health literacy to improve the health and well-being of all are some of Healthy People 2030’s overarching goals.^2^ Crucial milestones toward reducing health disparities include identifying and increasing awareness of differences among populations regarding health determinants and health outcomes.^1^

Within the United States, COVID-19 has disproportionately affected Spanish-speaking communities.^3–5^ This vulnerability can arise from many factors, including increased exposure, disproportionate burden of underlying comorbidities among racial/ethnic minority populations, and decreased access to health care, among other critical social determinants of health.^6^ Importantly, the lack of reliable and up-to-date information in Spanish has hindered attempts to combat the spread of COVID-19 in Hispanic communities.^7^ Adequately disseminating accurate health information to patient populations with limited English proficiency can bridge gaps in health care disparities.

Early in the pandemic, the rapid spread of inaccurate information led to an infodemic (epidemic of misinformation). Accurate information on COVID-19 illness was limited, particularly among traditionally marginalized communities.^8^

Hospitals are a trusted source of COVID-19 prevention and healthcare information for Spanish-speaking families.^9^ Initial efforts to combat the pandemic included rapidly disseminating COVID-19-related resources to the community. However, most of these resources were primarily in English. Following equity efforts, we sought to improve access to resources for our Spanish-speaking community. From May to August 2020, Spanish COVID-19-related resources on our institution’s website had an average of 2954 views per month compared with 48573 views per month to the English COVID-19-related resources, per our marketing and communications team’s Google Analytics tools. Self-reported admission data revealed preferred language was English and Spanish for 89% and 6% of our patients, respectively, reflective of the estimated 78.5% of the population in Greater Kansas City who prefer to speak English and 9.2% who prefer to speak Spanish.^10^ Due to fewer COVID-19 websites available in Spanish, the traffic to our Spanish websites should be disproportionately higher than our baseline percentage of the Spanish-speaking patient population. Our global aim was to increase awareness of and access to COVID-19-related resources for Spanish-speaking patients and families. Therefore, we aimed to close a gap in online traffic between English and Spanish COVID-19-related health information pages on our institution’s publicly-facing website by 50% within 10 months.

## METHODS

### Context

Children’s Mercy Kansas City (CMKC) is a free-standing, academic pediatric health system in Kansas City, Missouri, that cares for children mainly from Missouri, Kansas, but also from Nebraska, Arkansas, and Oklahoma. The self-reported preferred language of the population served by CMKC, as per admissions data (fiscal year 2020) includes: English (89%), Spanish (6%), and Arabic, Burmese, Somali, Vietnamese, and others (5%).

The hospital’s publicly-facing website contained pages with health information geared toward providers and patients on various topics. However, information in Spanish was lacking and required additional site navigation.

Of note, hospital staff provided routine updates to the community (as part of our city’s incident command center) via local news and social media/online platforms. Also, our hospital was a pediatric vaccine trial center led by a Spanish-speaking Infectious Diseases physician who had a presence within the community via press releases and interviews related to COVID-19 and vaccine trials. We cannot account for the impact these may have had on our measures.

### Multidisciplinary Team Formation

In response to growing evidence of disparities in COVID-19-related access to healthcare early in the pandemic,^11^ five native Spanish-speaking physicians entered our hospital’s process and performance improvement program. This training teaches solving problems in small group/team format through A3 improvement methodology, emphasizing a deep understanding of a system or process before carrying out improvement efforts.^12^

We formed an ethnically diverse, multidisciplinary team with a core team of five native Spanish-speaking physicians and ad hoc team members (information technology, marketing, public relations departments, and two process and performance improvement program coaches). Knowing that stakeholder input helps understand how families desire to receive and access COVID-19 information,^13^ we engaged with our hospital’s voluntary Patient and Family Advisory Council for Spanish-speaking families (“El Consejo”) throughout project design and implementation to elicit feedback on increasing access to our institutional Spanish COVID-19 resources. This feedback informed PDSA cycles and included the creation of resources and leveraging media outlets to expand reach.

### Planning the Interventions

**Fig. 1.**
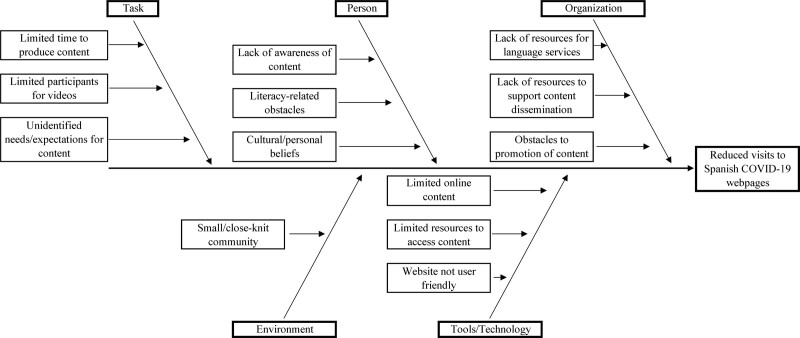
Cause-and-effect diagram.

Following the A3 methodology, we developed a cause-and-effect diagram where primary identified barriers were organizational (mainly lack of dissemination of existing resources and lack of available language services to improve existing resources), individual (lack of awareness of resources, literacy-related obstacles, cultural components related to trust in health information), and Environmental/Technological (small/closed community, lack of technological resources including electronic devices and stable internet connections; Fig. 1).

**Fig. 2.**
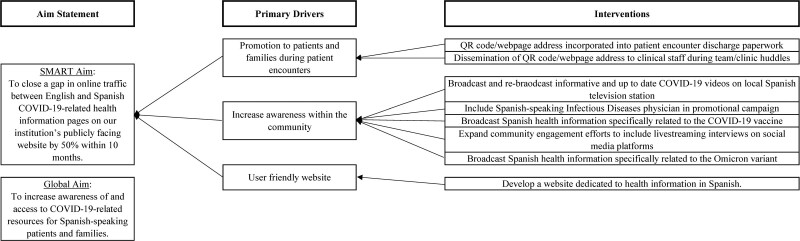
Driver diagram.

After investigating the system, we developed a key-driver diagram to guide interventions (Fig. 2). Primary drivers consisted of the promotion of Spanish COVID-19 resources within CMKC, increasing awareness in the community of up-to-date Spanish COVID-19 resources, and ensuring a user-friendly website was available in Spanish.

### Data Collection

Data were collected and displayed monthly based upon automated reports generated from online traffic to the CMKC publicly-facing COVID-19-related webpages in English and Spanish, as provided by our marketing and public relations department. Combined Spanish and English COVID-19 webpage traffic were included in the denominator for each outcome measure with respect to their specific tracking methodology, as outlined below (webpage views versus unique visits). Data were incomplete for 8/2021 due to data collection issues, so the 8/2021 data point includes only roughly half of the month.

### Outcome Measures

Our primary outcome measure was the monthly difference between total views of English versus Spanish COVID-19 webpages (total English COVID-19 webpage views minus total Spanish COVID-19 webpage views). We defined total webpage views as the number of times the webpage was loaded in a browser, regardless of user.

The secondary outcome measure was the monthly difference between total unique webpage visits to English versus Spanish COVID-19 webpages (total English COVID-19 webpage unique visits minus total Spanish COVID-19 webpage unique visits). We defined unique webpage visitsas the number of times a webpage was loaded within one session. Users returning to the same webpage several times during one session would only be counted once. This often serves as a proxy for the number of unique users.

A zero for either outcome measure would indicate identical traffic between English and Spanish webpages and unique visits. A positive value indicates greater traffic to English content, whereas a negative value indicates greater traffic to Spanish content.

Our goal was to reduce the difference in online traffic for each outcome measure by 50% within 10 months (9/2020 to 7/2021).

### Process Measures

Total monthly views and total monthly unique visits to Spanish COVID-19 webpages served as two of our process measures. Two similar process measures were tracked for English COVID-19 webpages. We chose these process measures because our interventions focused on the Spanish-speaking community, and monitoring these may signal special cause improvement before appreciating similar improvement in an outcome measure. For example, special cause improvement may be achieved in one of the process measures a few months before seeing special cause in the associated outcome measure (increases in English webpage traffic may occur at the same time as Spanish webpages, just at a slower rate).

Patient language data were unavailable at the outset of our project, so the above surrogate measures were developed. During postproject review, language data revealed that roughly 6% of our patient population preferred the Spanish language. With this information, a second set of outcome measures were developed to better understand impact. The first secondary outcome measure was the percentage of Spanish COVID-19 webpage views. The numerator was the monthly total views of Spanish COVID-19 webpages. The denominator was combined monthly total views to Spanish and English COVID-19 webpages. The second secondary outcome measure was the percent Spanish unique webpage visits. The numerator was the monthly total unique visits to Spanish COVID-19 webpages. The denominator was combined monthly total unique visits to Spanish and English COVID-19 webpages.

### Interventions

Leading up to our efforts commencing in 9/2020, and partly due to our team identifying a gap in available resources, CMKC’s communications and marketing department began translating a handful of high-priority English COVID-19-related webpages into Spanish. Yet, even by the time the project started, the volume of available webpages was insignificant compared with what was already available in English. Additionally, to allow the public quick access to reliable COVID-19 health information, an automatic dropdown header containing a link to English content had been implemented on the CMKC general homepage (www.childrensmercy.org). When we started our work, a paired dropdown header in Spanish was created linking to our institutional Spanish COVID-19 online resources.

Our team started with five PDSA cycles between 9/2020 and 5/2021 amd at increasing information dissemination via educational videos aired on the local Spanish television station, embedding a QR code within clinic depart paperwork, and sharing vaccine-related information. As we did not achieve our original goal within 10 months, three additional PDSA cycles were performed between 9/2021 and 1/2022, which included creation of a dedicated homepage for Spanish COVID-19 information^14^ and an additional educational video on the local Spanish television station regarding the Omicron variant (Table 1). We created an educational video showcasing one of the Spanish-speaking leaders of this project that aired on the local Spanish television station and was live-streamed on social media. The video informed our community about the website resources available in Spanish and reviewed accurate COVID-19 information, highlighting myths and misconceptions. The website in Spanish reflected the content of the COVID-19 in English website and was created by our marketing department.

**Table 1.** PDSA Cycles Dates and Descriptions

PDSA Cycle # (Date)	Description of Cycle
PDSA cycle 1 (9/2020 to 10/2020):	Leveraging our hospital’s connections, an educational video was created and aired on the local Spanish television station. The educational campaign informed our community about the website resources available in Spanish and aired 128 times during the cycle.
PDSA cycle 2 (11/2020):	To expand the reach to our providers, patients, and families, a QR Code/URL was developed which linked to the Spanish COVID website resources. This link was formatted in a manner to be included as part of the encounter discharge paperwork. This intervention was disseminated to clinical staff during morning team/clinic huddles spanning all levels of the organization.
PDSA cycle 3 (12/2020):	Building upon the previously aired advertisement, a new video was created and aired on the local Spanish television. This video featured an interview with a CMKC Spanish-speaking Infectious Diseases physician discussing up to date COIVD-19 information.
PDSA cycle 4 (1/2021):	A rerun of the original educational advertisement was again aired on the local Spanish television station to remind our community of the website resources available in Spanish.
PDSA cycle 5 (5/2021):	The website and local Spanish television station disseminated information on the Food and Drug Administration’s emergency use authorization for the COVID-19 vaccine in children aged 12–15 years. Emergency use authorization was granted on 5/10/2021.^13^
Although we had seen an early signal of improvement, we had not achieved our desired goal by 10 months. Therefore, additional cycles were implemented:	
PDSA cycle 6 (9/2021):	On 9/14/2021, our institution unveiled a separate, dedicated Spanish website with health information, beyond COVID-19 content.
PDSA cycle 7 (12/2021):	Partnering again with the local Spanish television station, another interview took place with a CMKC Spanish-speaking Infectious Diseases physician discussing up to date COVID-19 information, but this time the interview was live-streamed over social media.
PDSA cycle 8 (1/2022):	Given the winter surge of COVID-19 infections, an Omicron variant special educational video was aired on the local Spanish television station starting 1/13/2021.

### Study of the Interventions

Statistical process control charts assessed changes over time (QI Macros v2016.10, Know Ware International Inc., Denver, Colo.). Common and special cause variation were determined using previously established improvement rules.^15,16^

Project was deemed exempt as quality improvement by our institution’s Office of Research Integrity.

## RESULTS

There was a total of 1,226,196 combined webpage views with 369,983 views to Spanish COVID-19-related webpages [7188 baseline period (4/2020 to 8/2020); 362,795 implementation period (9/2020 to 1/2021)], and a total of 856,213 views to English COVID-19-related webpages (172,419 baseline period; 683,794 implementation period).

There was a total of 1,065,536 combined unique visits with 350,518 unique visits to Spanish COVID-19-related webpages (5691 baseline period; 344,827 implementation period), and a total of 715,018 unique visits to English COVID-19-related webpages (148,661 baseline period; 566,357 implementation period).

**Fig. 3.**
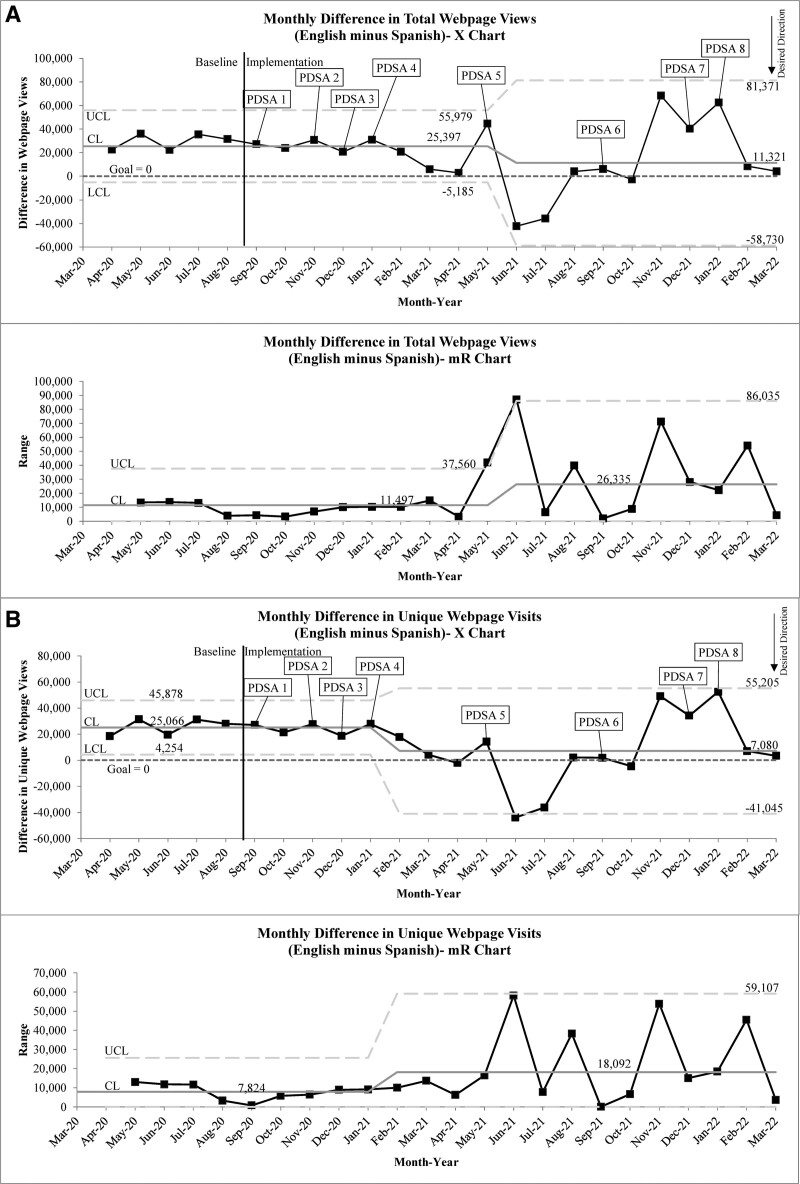
Outcome measures. A, Monthly difference in total webpage views. X-mR Chart. B, Monthly difference in total unique webpage visits. X-mR chart. A zero for either outcome measure would indicate identical traffic between English and Spanish webpages and unique visits. A positive value would indicate greater traffic to English content, whereas a negative value would indicate greater traffic to Spanish content. CL, centerline, LCL, lower control limit, UCL, upper control limit.

**Fig. 4.**
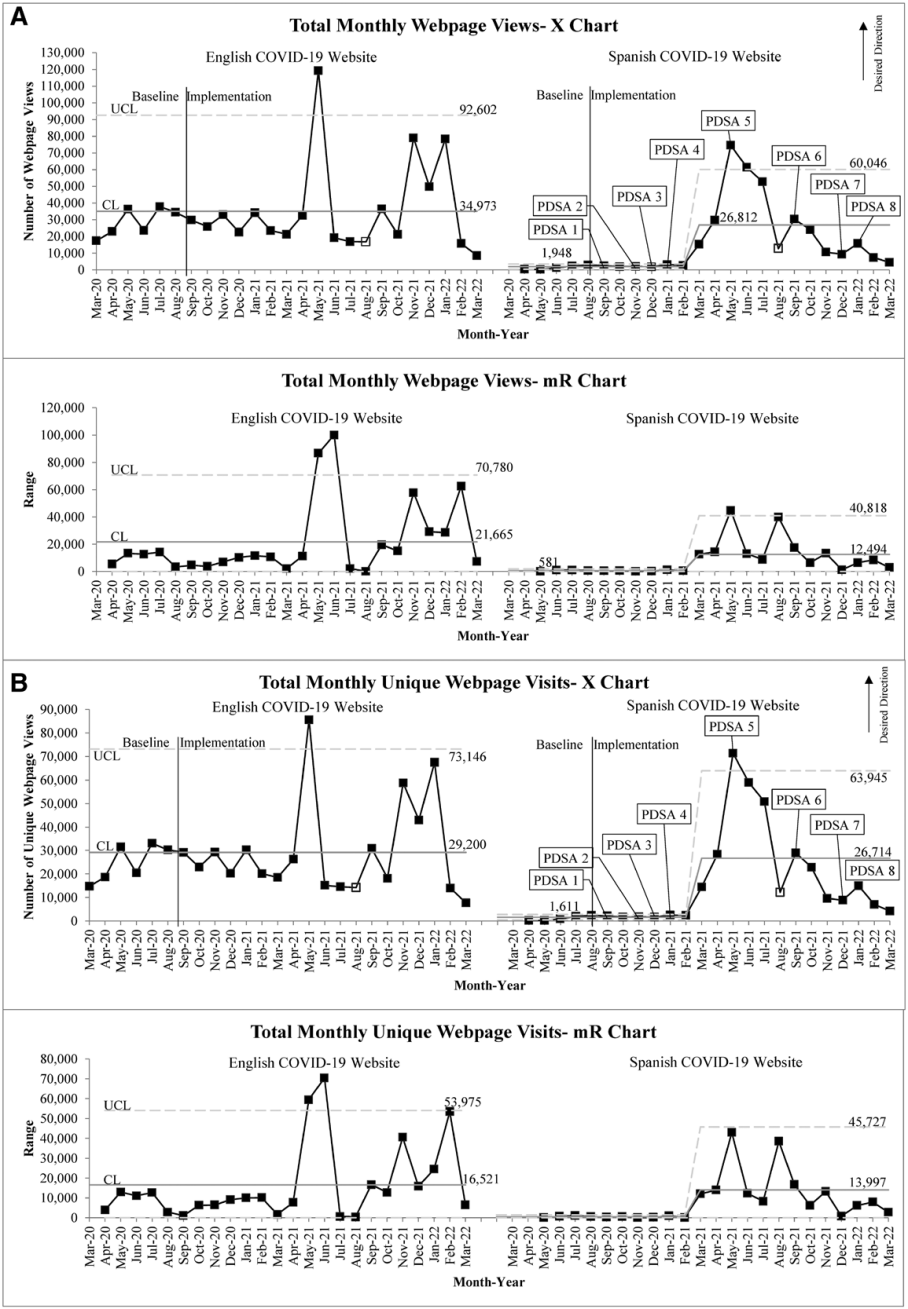
Process measures. A, Total monthly webpage views. X-mR chart. English on the left and Spanish on the right. B, Total monthly unique webpage visits. X-mR chart. English on the left and Spanish on the right. No Spanish data for March 2020. Partial data for August 2021 as indicated by open marker.

Special cause improvements in our outcome measures nearly mirrored one another, with astronomical data points occurring for both measures in June and July 2021. These data points immediately followed PDSA 5 (May 2021), and afterward, our system no longer displayed the same stability it had previously, suggesting a change in our system following May 2021 (Fig. 3). The second outcome measure showed slightly earlier special cause variation with a shift toward improvement starting in February 2021, where the June and July 2021 data points aided in meeting centerline shift criteria (Fig. 3B). Both outcome measures displayed sustained, special cause improvements in the centerline (mean) from a monthly difference of 25,397 to 11,321 webpage views (55.4% reduction, 6/2021, Fig. 3B) and 25,066 to 7080 unique webpage visits (71.8% reduction, 2/2021, Fig. 3B) corresponding to special cause improvements in the centerline (mean) of our Spanish webpage process measures (Fig. 4). Improvements were somewhat temporally associated with PDSA 5 (COVID-19 vaccine information) but appeared more to coincide with lead-up to and official emergency use approval of the COVID-19 vaccine for children aged 12–15 years (5/2021). This is supported by almost the entire increase in our Spanish webpage process measures being tied to a single website dedicated to post-COVID-19 vaccination instructions (>97% of traffic each month for 5/2021 to 7/2021; >90% for 5/2021 to 9/2021).

Improvements in our process measures also mirrored one another (Fig. 4). Spanish webpage process measures displayed an exponential increase in traffic for several months leading up to vaccine authorization, with peak traffic volume occurring in 5/2021. However, these had slow regression to the baseline in the centerline (mean) over a year. In contrast, English webpage process measures showed an astronomical data point in 5/2020 with subsequent data points returning to previous performance levels. The 5/2020 spike in English webpage process measures was not attributed to a single webpage, like the Spanish process measures but rather spread across various webpages.

Although not special cause, there were notable increases in traffic to English webpages from 11/2021 to 1/2022 (Fig. 4). These data points were driven primarily by traffic to the vaccine (11/2021) and testing (12/2021, 1/2022) webpages coinciding with emergency use authorization of the COVID-19 vaccine for children aged 5–11 years (10/29/2021)^17,18^ and the Omicron variant surge in our community (11/2021 to 2/2022). Similar increases were not seen in Spanish webpage process measures, however. Although PDSA cycles 8 and 9 were carried out in 12/2021 and 1/2022, respectively, they did not show notable improvements in our Spanish webpage process measures. Therefore, these cycles were not considered temporally-associated with changes seen in our outcome measures during the same period.

Review of our secondary outcome measures found sustained special cause improvement in both measures. Percent Spanish webpage views improved from a baseline of 6.2%–51.5% (March 2021) and regressed to 17.1% (November 2021) for the remainder of the project. (**See figure, Supplemental Digital Content 1,** which shows the Spanish COVID-19 webpage views—p Chart. http://links.lww.com/PQ9/A570.) Similarly, the percentage of Spanish unique webpage visits improved from a baseline of 5.8%–56.3% (March 2021) and regressed to 19% (November 2021) for the remainder of the project. (**See figure, Supplemental Digital Content 1,** which shows Spanish COVID-19 unique webpage visits—p Chart. http://links.lww.com/PQ9/A570.)

## DISCUSSION

Using the A3 improvement framework^19^ our multidisciplinary team, led by five native Spanish-speaking physicians from an underrepresented minority group, exceeded our aim of reducing the difference in online traffic between our institution’s English and Spanish COVID-19-related webpages by more than 50% within 10 months (9/2020 to 7/2021). Most interventions focused on increasing the public’s awareness of and access to our institutional Spanish COVID-19 webpages. Interestingly, these interventions in themselves did not seem to drive improvement but rather, circumstances outside of our team’s control exerted greater influence on our measures (ie, emergency use authorization of vaccines and surge in Omicron variant). However, our efforts helped drive traffic to our webpages in times of high demand for linguistically appropriate services (pediatric vaccine roll-out-related uncertainty).

When the COVID-19 pandemic unfolded, and the infodemic took hold, patients and families were searching for reliable pediatric information. Our first steps involved iterative improvement cycles focused on updating and increasing awareness of current Spanish COVID-19-related resources on our hospital’s website. Although our interventions did not directly show improvements in our measures, we proved to our community that CMKC was also a trusted source of pediatric COVID-19 health information.

We observed marked improvements in website traffic when COVID-19 vaccines became available for patients aged 12–15 years and again for patients aged 5–11 years, suggesting an ingrained knowledge about the existence of resources on our website available in the language of interest. Increasing the dissemination of accurate and updated health information on COVID-19 to Spanish-speaking families can help decrease health disparities and the burden of this disease. In addition to addressing a longstanding inequity across the US healthcare landscape, combating disparities during the pandemic can lead to better health outcomes for the entire population.

Historically, dissemination of healthcare information during pandemics first reaches communities already connected with the health system, thereby increasing gaps in already existing health disparities.^20,21^ Previous literature has described several promising practices to disseminate public health information among non-English speaking communities including (1) community engagement throughout the project, (2) dissemination of translated messages, and (3) communication through culturally appropriate and trusted channels.^22^ These approaches in isolation may not have been effective, but they worked synergistically, resulting in improvement in the outcome measures. Our experience builds on prior literature and offers novel insight into the potential impact of engaging a multidisciplinary team of Spanish-speaking physicians in understanding obstacles to health information dissemination to the Hispanic community. Our work also strengthens the literature base that improvement methodology can be utilized to improve the dissemination of language-appropriate health information to patients and their families while attempting to close disparity gaps.

Several unintended benefits occurred. First, although not formally tracked, increased traffic through our COVID-19 webpages may have advanced knowledge of this disease among patients and their families who belong to other racial and ethnic minority populations. Second, it allowed improvement of the navigability of our institution’s general publicly-facing website, mainly to rapidly find COVID-19 information in Spanish, and helped our institution understand the benefits in and ease of implementation of QR code embedded health information incorporated into the clinical encounter discharge paperwork. Third, and although not measured, in holding multiple conversations on improving information dissemination, the project might have increased awareness among our hospital staff and colleagues of COVID-19 health disparities. Lastly, through geolocation, we learned that our Spanish COVID-19 webpages were being accessed by individuals in other countries, particularly Mexico, suggesting our efforts may have resulted in an international reach.

### Limitations

First, the project was conducted at a free-standing, academic children’s hospital in the Midwest, surrounded by growing Hispanic communities.^23^ Although lessons learned may be applied to other healthcare settings and communities, generalizability may be limited. Second, the project focused specifically on Spanish COVID-19 information rather than including all health information or other languages and thereby may have exacerbated other language-based disparities unintentionally. Third, we did not have a specific patient-centered outcome. More proximate outcomes to be considered include increase in knowledge regarding COVID-19 or change in intention to take preventive actions. Also, although we engaged our hospital’s Patient and Family Advisory Council for Spanish-speaking families ““(“El Consejo” ““) throughout project design and implementation, we did not monitor patients and “families” satisfaction.

Additionally, we did not have health literacy specialists on the multidisciplinary team. Also, increased use of content does not equal accessibility, understandability, or acceptability of the web content. It might not be associated directly with increased knowledge, as it could even have adverse effects. Finally, increased use of the content does not automatically reflect interaction with the content, in general, or by the target community.

Future directions could address limitations, including enhancing understanding of obstacles to sustained improvement, further inclusion of key stakeholders, and utilization of new platforms for public health information dissemination, such as social media and virtual conferencing.

## ACKNOWLEDGMENTS

The authors would like to thank our hospital’s process and performance improvement program, “Problem-Solving for Teams” supported by Children’s Mercy Berry Institute. We would also like to thank Leesa Brown for formatting and collating data, our Communications and Marketing Department (specifically Mistie Young, Sarah Young and Jennifer Silvers), as well as Lucia Zamarron (Language Services), Megan Bolton (Health informatics and Tech) and Deejo Miller (Patient and Family Engagement/Office of Patient Experience) who together with the Patient and Family Advisory Council for Spanish speaking families (“El Consejo”) made this work possible. This study did not require ethics approval.

## Supplementary Material


