# Supplementary material for: Combating Disparities in a Pandemic: Increasing Dissemination of Coronavirus Disease 2019 Resources in Spanish
Source: Pediatr Qual Saf. 2024 Jul 10;9(4):e744. doi: 10.1097/pq9.0000000000000744 (PMC11236400; doi:10.1097/pq9.0000000000000744)
Supplement: Supplementary file 1 [file pqs-9-e744-s001.pdf]

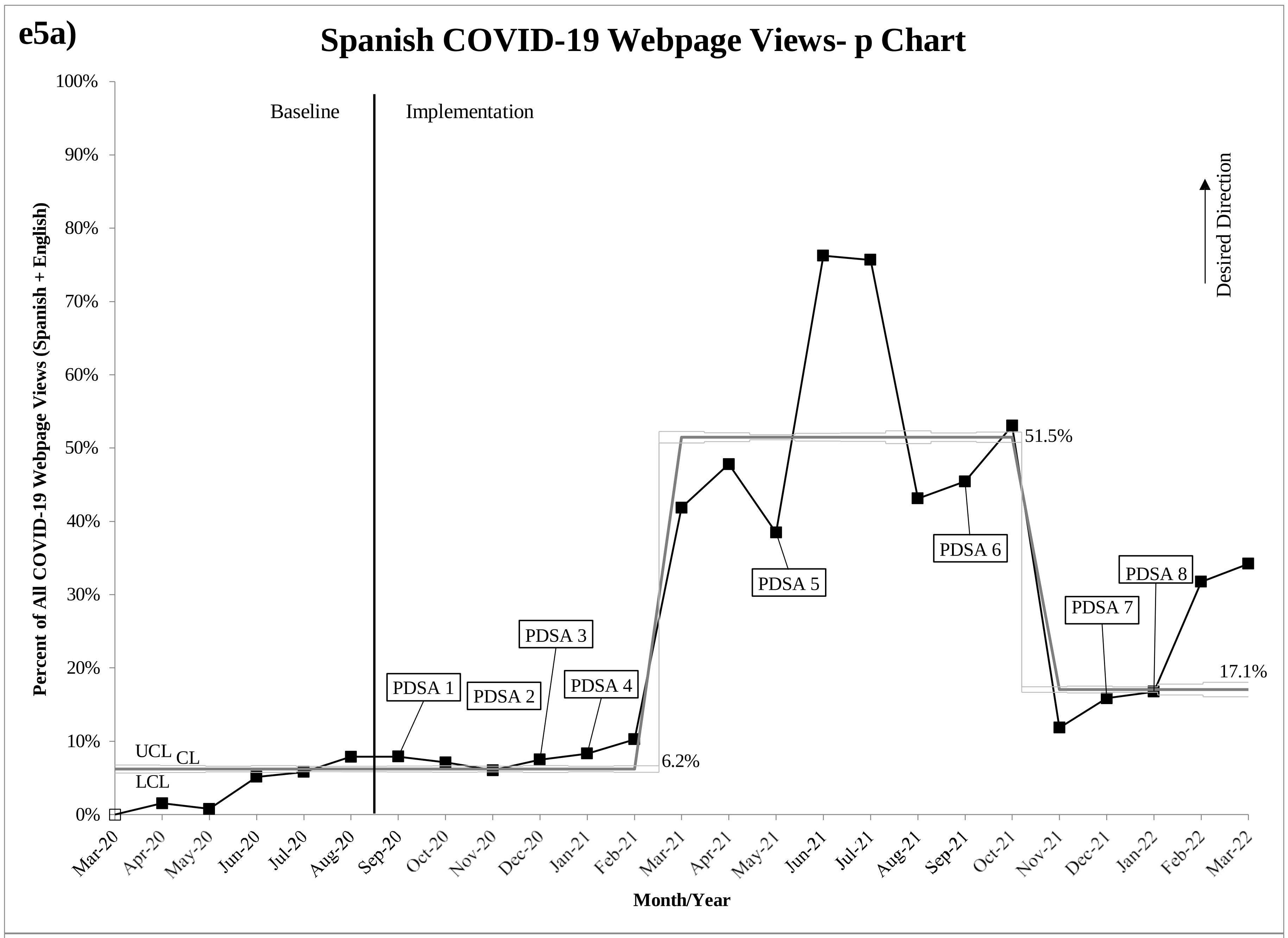

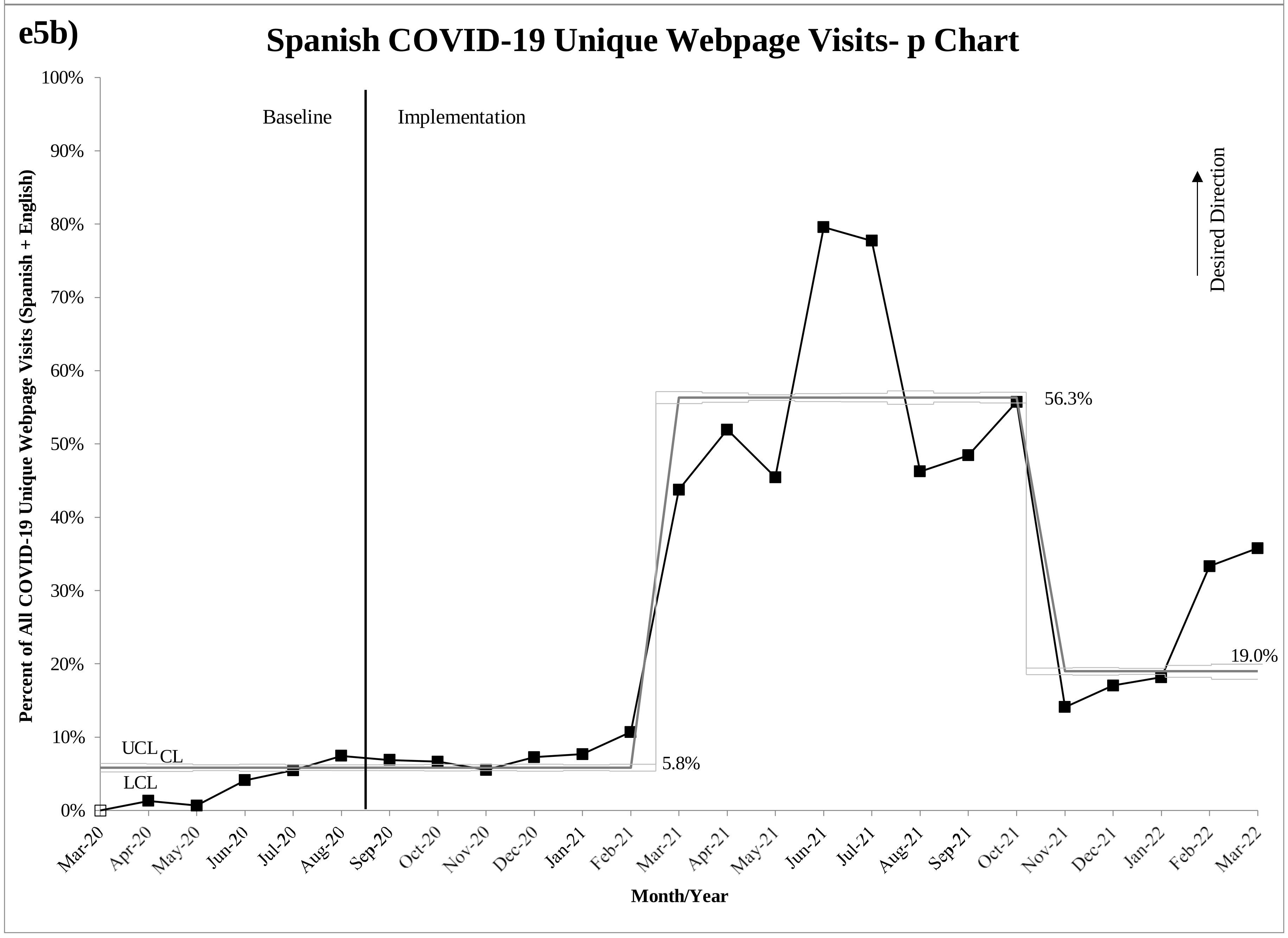

eFigure 5. Secondary Outcome Measures.

eFigure 5a. Monthly Percent of Spanish COVID-19 Webpage Views. p Chart.

eFigure 5b. Monthly Percent Spanish COVID-19 Unique Webpage Visits. p Chart.

Each figure represents the percentage of views (or unique visits) to Spanish COVID-19 webpages out of total views (or unique visits), respectively. Total views (or unique visits) was determined by combining total Spanish webpage views (or uniqu visits) plus total English webpage views (or unique visits). March 2020 data point 'ghosted' due to no Spanish webpage data available. CL, centerline, LCL, lower control limit, UCL, upper control limit.
